# “A place to have fellowship”: implementing group counseling in a clinical fellowship program

**DOI:** 10.1186/s12909-026-09159-x

**Published:** 2026-04-07

**Authors:** Anoushka Sinha, Sara M. Buckelew, Marissa Raymond-Flesch

**Affiliations:** 1 Division of Adolescent and Young Adult Medicine, Department of Pediatrics, University of California San Francisco, Box 0110, 550 16th St, San Francisco, CA 94158 USA; 2 Division of Medical Education, Department of Pediatrics, University of California San Francisco, San Francisco, CA USA; 3 Philip R. Lee Institute for Health Policy Studies, University of California San Francisco, San Francisco, CA USA

**Keywords:** Fellowship, Training, Group counseling, Mental health

## Abstract

**Background:**

The Accreditation Council for Graduate Medical Education (ACGME) has endorsed recommendations to promote individual counseling services to support trainees. However, there are limited data and no clear recommendations related to group counseling services, a format that may be scalable and support peer connection. This paper has two primary objectives: (1) to describe the implementation of group counseling as a method of providing formative, normative, and restorative supervision to GME trainees and (2) to report participants’ experiences within the program.

**Methods:**

In September 2019, the fellowship program implemented regular group counseling for fellows, with ongoing feedback collected to optimize logistics. Between November 2023 and January 2024, we worked within an interpretivist paradigm to explore experiences with group counseling by conducting semi-structured individual interviews with former and current fellows, which were transcribed and analyzed thematically. This study was deemed exempt from review by the Institutional Review Board.

**Results:**

Eleven out of 13 current or former fellows (85%) participated in the interviews. Participants described the challenges of their training and perceived benefits of group counseling related to well-being, connection, and professional identity development. They offered the following recommendations for group counseling: clear confidentiality guidelines, protected time during work hours, facilitation by a mental health clinician based outside the training program, and optional attendance.

**Conclusions:**

This study suggests that professionally facilitated group counseling may offer perceived benefits for trainees. Further research is needed to address attendance challenges and explore strategies for integrating group counseling into different training programs.

## Background

The convergence of a growing number of distressed patients and a depleted healthcare workforce has reshaped the clinical challenges and learning needs of graduate medical education (GME) trainees. [1–6] To support these trainees, the ACGME requires access to confidential, affordable mental health assessment, counseling, and treatment [7]. Reports in the literature demonstrate how this can be accomplished by providing individual counselingm [8, 9]. However, there is limited evidence and limited practical guidance on how to implement professionally facilitated group counseling for GME trainees, as well as limited qualitative understanding of how trainees experience such programs [10–13].

The University of California San Francisco (UCSF)’s Division of Adolescent and Young Adult (AYA) Medicine fellowship program offers comprehensive training in primary care and managing complex medical conditions, including attention and mood disorder management, sexual and reproductive health, gender care, eating disorders, and addiction treatment. Reflecting national trends of rising adolescent admissions [14, 15], the division’s average inpatient census began rising in 2017 and had quadrupled by 2020. The increased patient volume and concurrent growing mental health acuity highlighted the need for additional resources to support trainees.

In response to these growing concerns, the program introduced group counseling for fellows incorporating Proctor’s model of clinical supervision, a useful framework from nursing and mental health services training that may be applied to ACGME training programs. Proctor’s model is notable for including formative supervision (focusing on skill development, knowledge acquisition, and professional identity growth), normative supervision (supporting the maintenance of standards of practice and care), and restorative supervision (aimed at enhancing well-being, reducing burnout, and improving job satisfaction) [16, 17] This framework is especially applicable to GME, where rapid professional identity formation occurs amid long hours and high clinical demands that can compromise well-being.

This paper has two primary objectives: (1) to describe the implementation of group counseling as a method of providing formative, normative, and restorative supervision to GME trainees and (2) to report participants’ experiences within the program.

## Methods

### Development and implementation of group counseling

In September 2019, the fellowship program partnered with UCSF’s Employee Assistance Program (EAP) to introduce a biweekly counseling group for fellows. UCSF’s EAP offers confidential individual counseling to UCSF faculty, staff, residents, postdocs, and clinical fellows free of charge, with providers specializing in organizational psychology. The fellows’ counseling group was the first long-term counseling group for trainees facilitated by the EAP. The process for addressing any serious mental health concerns or fitness-for-service issues raised during group counseling mirrored the EAP’s standard protocol for any employee seeking mental health services.

In 2019, the fellowship program director met with the EAP director to outline the goals of the counseling group, which included (1) providing restorative support to address stress and support fellows’ well-being, (2) offering formative consultation to facilitate reflection and clinical skill development, such as managing transference and countertransference, and (3) emphasizing norms that maintain standards of practice and care. Program leadership maintained that participation in counseling should remain voluntary, with efforts focused on ensuring it was accessible, feasible, scheduled as an opt-out activity, and free of charge for fellows, aligning with ACGME requirements for mental health care access [7].

Per the EAP director’s preference, two psychologists from the EAP were selected as the initial facilitators as is common in group therapy settings. Although facilitators originally proposed 90-minute sessions, program leadership and fellows agreed on one-hour sessions to better accommodate schedules. The fellowship program ensured fellows were relieved from clinical duties and administrative tasks to attend these sessions. With the onset of the COVID-19 pandemic in mid-2020, the EAP had to suspend its facilitation due to other pandemic-related responsibilities. During this period, two university psychotherapists with no regular contact with pediatrics trainees were hired to lead the group until the EAP facilitators could resume their roles seven months later. Program leadership gathered feedback from fellows and facilitators every one to three months to fine-tune group logistics. From 2019 to 2023, approximately 40 sessions were held.

The sessions followed a format that incorporated formative, normative, and restorative supervision by featuring an opening mindfulness activity, a self-reported burnout check (on a scale from 1 to 10), an unstructured open discussion that included sharing skills, expectations, and advice, and a closing exercise. After consulting with the fellows, facilitators decided not to use a manual guide for the sessions, allowing flexibility in addressing participants’ needs. Before the pandemic, sessions were held in person at an academic building on campus, but since 2020, they were conducted over Zoom. The timing of the sessions varied depending on the availability and preferences of both fellows and facilitators. Facilitators consistently reinforced the terms of confidentiality, reminding fellows to maintain discretion when discussing group matters outside the sessions and only to share information with others’ explicit consent. As in any counseling group, fellows were encouraged to provide peer support. Faculty members, including program leadership, were never present during the counseling sessions to maintain a confidential and supportive environment.

### Qualitative interviews with group participants

We worked within an interpretivist paradigm to explore fellows’ subjective experiences with group counseling. The research team included a current fellow (AS), the fellowship program director (MRF), and a senior faculty member (SB) from the division. AS and MRF developed the semi-structured interview guide deductively, targeting areas of interest informed by the study aims, and inductively identified additional themes from the interviews.

AS sent email invitations to all AYA fellows who had participated in the counseling groups to take part in 30-60-minute individual interviews. Participants were informed about the study’s purpose, procedures, and their rights, and verbal consent was obtained prior to participation; participants were not compensated. Between November 2023 and January 2024, AS conducted the interviews over Zoom, transcribed them verbatim, de-identified the transcripts, and shared them with the research team.

Analysis followed a primarily inductive approach, with sensitizing concepts from Proctor’s model informing interpretation. AS and MRF first reviewed interview transcripts and discussed emergent themes through iterative analytic memoing and team meetings, which informed development of a shared codebook. Initial codes reflected topics included in the interview guide (e.g., well-being, peer support), with additional codes generated inductively. MRF assisted with and reviewed coding of initial transcripts to ensure fidelity to the codebook, with SB providing input to resolve differences in interpretation. Following this process, AS completed coding of the remaining transcripts using Dedoose. After the seventh interview, analysis suggested diminishing returns of new themes; remaining participants were interviewed to ensure representation across training stages. This study was deemed exempt from review by the Institutional Review Board.

The idea for this study was generated by AS, whose position as a current fellow may have facilitated rapport and trust with participants. At the same time, the research team recognized that insider status and program leadership involvement could shape what participants felt comfortable disclosing. To address these considerations, program leadership did not recruit or interview participants, transcripts were de-identified prior to team review, and MRF and SB were not informed of participants’ identities. Participants were invited to review and comment on the study findings to assess resonance with their experiences. MRF’s experience in stakeholder-engaged and community-based participatory research informed the study design and aligned the research process with the program’s ongoing efforts to incorporate fellow feedback into program improvement.

## Results

All 13 current and former fellows participated in at least one counseling session. Of these, 11 agreed to be interviewed, representing 85% of the group, with demographics consistent with the overall composition of the fellowship program (Table 1).

**Table 1:** Sociodemographic characteristics of participants (*n* = 11)^a^

Demographics	*n* (%)
Gender	
Female	8 (73%)
Male	3 (27%)
Race	
Asian	3 (27%)
White	8 (73%)
Ethnicity	
European/European Descent	7 (64%)
Other Ethnicities^b^	4 (36%)
Career Path *(may choose more than one)*	
Academic	10 (91%)
Clinical	9 (82%)
Education	11 (100%)
Research	7 (64%)

^a^Three participants (27%) volunteered identifying as LGBTQ

^b^Further details not provided to protect participants’ identities

One current fellow declined to participate, and another was on leave when the interviews were conducted. Although attendance fluctuated, programmatic support to protect fellows’ time generally resulted in participation from approximately two-thirds of fellows per session. For confidentiality reasons, individual attendance was not tracked, which prevents evaluation of engagement and dose response (i.e., how many sessions might be needed to derive benefit).

### Qualitative data analysis

**Fig. 1:**
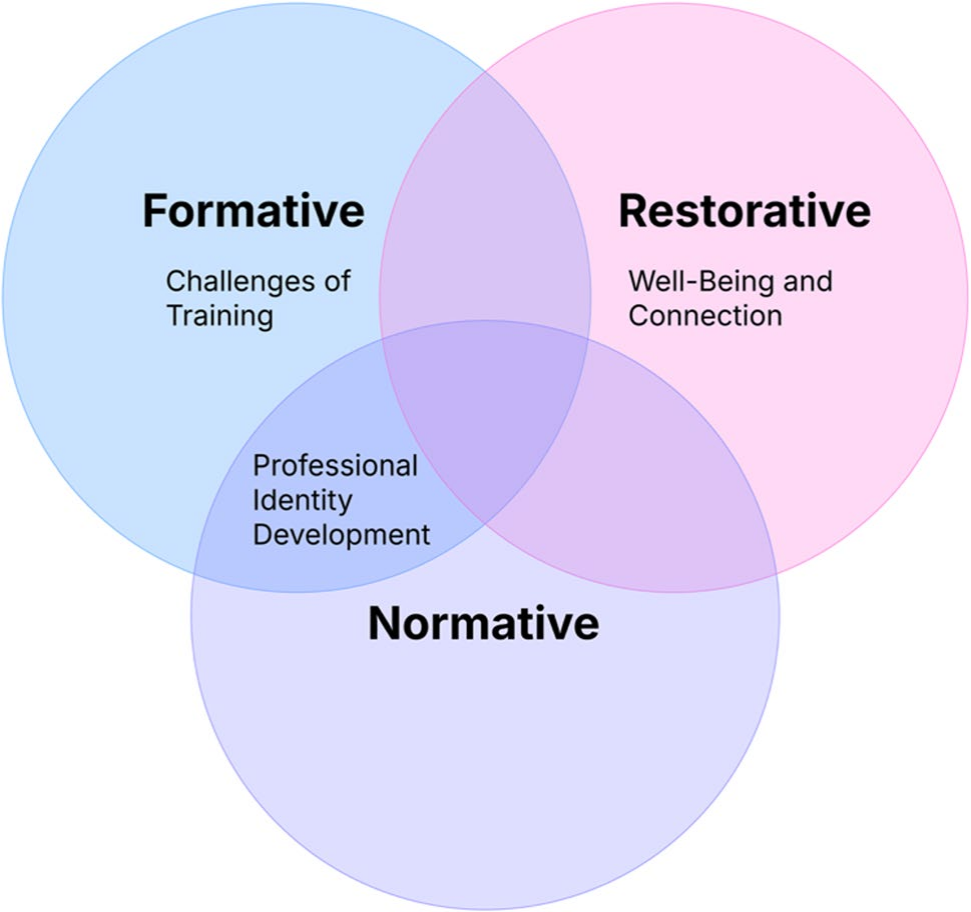
Alignment of emergent themes with Proctor’s model

Participants described their experiences with group counseling across clinical, relational, and professional domains. Twenty-two initial codes were grouped into 13 sub-themes and four overarching themes in accordance with Proctor’s model (Fig. 1):

Challenges of Training, Well-Being and Connection, Professional Identity Development, and an additional category of Logistics (Table 2).

**Table 2:** Themes and exemplar quotes

Themes	Exemplar quotes
Challenges of training	*Things really specific to the field that we do come up: really challenging patient encounters*,* really scary…encounters*,* and ones that are just lingering in your head and beat you up a lot.* *I feel like a lot of time [was] thinking about those ongoing challenges that are going to come up with any type of psychiatric diagnosis and that it’s not always as cut and dry as other things in medicine.*
Well-being and connection	*It takes away any stigma from accessing…a confidential space to work through some of those challenges…and just to normalize some of the things that are hard.* *[Group counseling] helped us put into words the feeling that we were experiencing*,* helped us feel connection with our other fellows who actually were in the same boat*,* and we didn’t feel scared to kind of talk about our experiences.*
Professional identity development	*I want to integrate some of those principles in terms of debriefing and a safe space…and seeing it done in a very structured way*,* and so those are some of the principles I’m hoping to carry forward with me.* *I learned things that I use in my professional career like…how to ask questions and how to reframe things and how to keep things open-ended.*
Logistics	*I would say confidentiality is key. I would say ownership among the fellows for the agenda is also key.* *Before offering or mandating this universal service*,* it’s important to ask what people really need out of a support group.*

### Challenges of training

This theme aligned most closely with the *formative* function of Proctor’s model. Overall, the two most predominant sub-themes related to this theme were navigating the intensity of AYA clinical training and structural gaps in team-based support during training.

### Navigating the intensity of AYA clinical training

Most participants emphasized that group counseling was particularly relevant to their training in AYA medicine. F2 noted:

Our fellowship I think, is really tough compared to a lot of other fellowships out there just by the nature of the complex psychosocial patient care that we do. And so, a lot of it was about having the space to listen, to vent, to unload, to process, and to problem-solve.

Participants described caring for AYAs at key developmental stages, often in the context of serious illness, which heightened emotional labor and blurred personal-professional boundaries.

### Structural gaps in team-based support during training

The lack of adequate multidisciplinary support, especially during the pandemic, further intensified the challenges that fellows faced. F9 said:

Without any psych support or social work support, more being loaded onto the fellows…it just felt untenable in some ways, like we were having a lot of fellows feeling like we couldn’t continue, and it felt really tough, and there was a lot of burnout in a really acute period of time…It was such an intense time, and it just felt like things were spiraling.

Given the increasing volume and complexity of patients, fellows appreciated having dedicated group counseling to process the difficulties of their training alongside peers experiencing similar struggles.

### Well-being and connection

This theme mapped onto the *restorative* function of Proctor’s framework. Most interviewed fellows expressed appreciation for group counseling and supported its continuation. While experiences varied by timing, rotation demands, and individual readiness, participants consistently described peer witnessing, normalization of distress, and developing strategies for self-care as salient aspects of the group counseling experience.

#### Peer witnessing as a source of emotional support

Most participants described perceived improvements in well-being and connection with fellows, often on different rotations. The sessions included discussions of challenging patient cases, problem-solving related to the fellowship experience, and realizing that their struggles were not isolated. F2 said that the sessions “provided not only an outlet to…discuss and process a lot of the really challenging cases we were seeing but also a place to have fellowship…in the true definition of being around five other people who were going through the same things that I was going through and to hear from them.”

#### Normalization of distress as stigma reduction

Beyond providing immediate peer support, the group sessions appeared to influence fellows’ attitudes toward seeking mental health care more broadly. Several fellows even sought individual therapy after participating in the group sessions. F1 said, “It takes away any stigma from accessing…a confidential space to work through some of those challenges…and just to normalize some of the things that are hard.”

This reframing of distress as a common and expected aspect of training helped reduce perceived stigma around therapy and encouraged fellows to access additional support when needed.

#### Developing strategies for self-care

Participants highly valued the communal validation that they experienced in the group therapy and expressed appreciation for the opportunity to learn and develop self-care strategies through the sessions. F10 stated:

The psychologist really did offer some potential tools…For example, giving yourself grace. Doing things even during call that are…self-care, in a sense that you’re still important and you need to take care of yourself in order to be the best version of yourself on call… So, it was very practical tools that I’ve actually implemented, and I think have been helpful.

Participants noted that hearing peers describe similar struggles reinforced the legitimacy of these practices, making it easier to adopt self-care strategies without feeling disengaged from work responsibilities. However, engagement with the groups varied, and some fellows chose not to participate at times as part of attending to their own needs. As F3 explained, “When you’re there, and you need it, and it’s helpful, and it’s working, and you’re vibing with people, it’s great, but if you’re not in that headspace, it can feel a little bit less productive.”

### Professional identity development

This theme aligned most closely with the *formative* and *normative* functions of Proctor’s framework. Sub-themes included mentorship as a professional responsibility, transition from trainee to faculty clinician, and professional identity shaping approaches to patient care.

#### Mentorship as a professional responsibility

Several participants highlighted professional identity development, mainly as they learned to mentor other fellows throughout their training. F8 said, “I want to integrate some of those principles in terms of debriefing and a safe space…and seeing it done in a very structured way…those are some of the principles I’m hoping to carry forward with me.”

#### Transition from trainee to faculty clinician

Participants noted that the opportunity to guide trainees at earlier stages of fellowship marked a significant shift in their professional identity, which continued into their faculty roles. F1, who was a faculty member at the time of the interview, said:

I think now as faculty [about] being thoughtful about how different challenging scenarios can impact my trainees, right? And even just myself. And the need to, you know, check in with people…even with, you know, my colleagues–about different obstacles that come up along the way and kind of the role that that can play.

Participants described experiences they associated with developing leadership skills by fostering a deeper understanding of their own needs and the needs of other trainees, which they viewed as preparation for future faculty roles. F7 described:

It was an opportunity for me to grow in my own self-awareness, which was super helpful and key in making that transition from being a trainee to an attending. That ongoing work at self-reflection and trying to be aware of what my needs are and taking care of myself were skills that I definitely practiced a lot as a fellow.

#### Professional identity shaping approaches to patient care

Participants also noted that the benefits of professional identity formation translated into improvements in their patient care. F2 mentioned that being a part of group counseling provided an essential lesson in aligning her practice with the advice she offers to patients, enhancing her ability to care for them more effectively: “All therapists have to have therapy of their own. In many ways, we also need to learn some of the things that we prescribe in order for us to kind of be genuine in our practice.”

### Logistics

Several common points emerged from the interviews concerning the logistics of implementing group counseling, which were related to confidentiality, format, timing, facilitation, and voluntary attendance.

#### Confidentiality

Confidentiality was universally recognized as essential, providing a sense of safety that enabled shared vulnerability and trust during the sessions.

#### Format

Most participants preferred in-person meetings, though one participant noted that having a virtual option benefited those off campus. Indeed, one participant chose to join sessions via Zoom while on leave, allowing them to stay engaged with colleagues.

#### Timing

Most participants recommended that the sessions be held monthly or on-demand, that faculty be informed to ensure this time is protected, and that the sessions occur during work hours, avoiding lunchtime. As F8 described, “Oftentimes I’m literally at the cafeteria because it’s always during lunch break, and so it’s not the time or the place for me to really benefit.”

#### Facilitation

Participants also emphasized the importance of selecting an appropriate facilitator. The consensus was that the facilitator should be a mental health clinician from outside the training program with experience working with trainees. Participants valued facilitators who not only listened but also provided practical tools and skills that they could apply in their work. Additionally, participants expressed a desire to offer feedback on facilitators to ensure they were a good fit for the group. When available, the involvement of internal EAP providers was appreciated for providing continuity and institutional knowledge.

#### Voluntary attendance

Most participants emphasized the importance of keeping session attendance voluntary. Several noted that there were times when they did not feel inclined to engage in counseling. As F10 explained, “If I was in a good headspace, it might bring me down.” However, variability in attendance occasionally disrupted the group dynamic and appeared to limit the benefit participants could derive from the sessions. F5 said:

For better or for worse, I do feel like support groups work best when everyone buys into it. Making something like that mandatory is also not OK. But I think being more attuned to figuring out what people actually want and need and then trying to tailor something to that in order to get buy-in from every single person would be helpful.

Overall, participants expressed a desire for group counseling that evolves to meet fellows’ needs, with ongoing refinement by program leadership and facilitators to support well-being and professional identity development.

## Discussion

We have described the implementation of group counseling using Proctor’s model of clinical supervision, which includes formative supervision to target skill development, normative supervision to maintain standards of practice and care, and restorative supervision to reduce burnout; the restorative component is not typical of other clinical supervision models, which is why we chose it [16, 17]. Leveraging Proctor’s model in group counseling may support fellows’ experiences of well-being, connection, and professional identity development. While prior studies have shown that peer support can be beneficial for trainees, they are often limited by relying on peer or near-peer facilitators rather than trained mental health clinicians [11–13], who may lack social congruence but are equipped to provide nuanced therapeutic support (e.g., psychological containment, therapeutic skills, managing group processes, and advanced reflective supervision techniques). Our exemplar offers a promising implementation strategy for supporting trainee well-being. With rising mental health concerns affecting GME trainees across various disciplines, this format warrants further exploration as a scalable intervention.

Participants’ recommendations align with commonly described practices in group therapy—such as explicit confidentiality [18], protected time during work hours [19], and facilitation by a mental health clinician based outside the training program, practices which do align with the ACGME requirements [7]. Participants also emphasized that group counseling should remain optional, though this presents a challenge: inconsistent attendance could disrupt the group dynamic, and a critical mass may be necessary to justify the involvement of a mental health professional. Further research is needed to explore how to balance voluntary attendance with the need for steady attendance to ensure the cohesion and effectiveness of group counseling. GME programs should seek feedback from trainees on optimal group size, and we recommend including a mix of fellows from different class years to enhance peer support and mentorship.

Limitations of this study include generalizability, as AYA fellows experience unique stressors, and trainees in other fields may have different stressors and may experience group counseling differently. In addition, fellows are in a more advanced stage of professional identity formation, so the dividends of group counseling may differ for trainees in residency programs. The demographics of participating fellows (Table 1) reflect the composition of one AYA Medicine fellowship program and may not represent trainees across other GME programs or institutional contexts. Limited racial and ethnic diversity within the sample constrains the applicability of findings to trainees from marginalized or underrepresented backgrounds. Future studies should include more diverse trainee populations to examine how group counseling experiences and perceived benefits may vary across identities and training environments.

The interviewer’s insider status as a current fellow may have facilitated rapport and candid discussion but may also have shaped what participants felt comfortable disclosing. Similarly, program leadership co-authorship may have introduced social desirability pressures despite safeguards. To mitigate these risks, leadership did not recruit or interview participants, transcripts were de-identified prior to team review, and participants were explicitly invited to share both positive and negative feedback. Analytic decisions were discussed across the research team with attention to discrepant or mixed perspectives when present; however, the small sample limits the extent to which divergent experiences could be fully explored.

The absence of attendance tracking limits assessment of engagement and the relationship between intervention “dose” and perceived benefit. This study does not measure objective outcomes and therefore conclusions should not be framed as evidence of effectiveness. Larger qualitative studies in the future could provide additional data to assess saturation more robustly and explore additional sub-themes across diverse training contexts.

These limitations notwithstanding, our exemplar is novel for implementing group counseling that incorporates Proctor’s model of clinical supervision, with perceived benefits in well-being and professional identity formation. Further research is needed to assess the experiences of facilitators, the broader impact of group counseling, and the optimal structure of this practice for various training environments.

## Conclusions

Medical training and clinical care are often rife with medically complex and emotionally demanding situations. With growing interest in mental health and well-being in training institutions, particularly in response to widespread burnout, there is a pressing need for guidelines and evidence to inform best practices for providing psychological support for GME trainees. As demonstrated by this qualitative study, group counseling represents a potentially valuable approach for supporting reflection, connection, and professional identity development among trainees. These findings offer practical guidance for programs considering professionally facilitated group counseling while highlighting the need for further research to evaluate outcomes, optimal structure, and broader applicability across training contexts.

## Data Availability

The datasets used and/or analyzed during the current study are available from the corresponding author upon reasonable request.

## Abbreviations

ACGME: Accreditation Council for Graduate Medical Education
AYA: Adolescent and Young Adult
EAP: Employee Assistance Program
UCSF: University of California San Francisco
